# Effects of plyometric training on balance, joint position sense, and explosive strength in young taekwondo athletes

**DOI:** 10.1186/s13102-026-01762-1

**Published:** 2026-05-22

**Authors:** Ahmet Mücahit Özcan, Ali Osman Kıvrak

**Affiliations:** Department of Coaching Education, Faculty of Sport Sciences, Selçuk University, Konya, Türkiye

**Keywords:** Explosive strength, Balance, Joint position sense, Plyometric training, Taekwondo

## Abstract

**Background:**

The purpose of this study was to investigate the effects of plyometric training on balance, joint position sense, and explosive strength in young taekwondo athletes.

**Methods:**

A total of 20 taekwondo athletes aged between 15 and 18 years voluntarily participated in the study. Participants were randomly assigned to an experimental group (*n* = 10; 5 females, 5 males) and a control group (*n* = 10; 5 females, 5 males). In addition to regular taekwondo training, the experimental group performed a plyometric training program three times per week for eight weeks, while the control group continued only their regular training. A 2 × 2 mixed-design (group × time) was employed. A two-way repeated measures ANOVA was conducted to examine the main effects of group (experimental vs. control) and time (pre-test vs. post-test), as well as the group × time interaction effect.

**Results:**

The Significant group × time interaction effects were observed for eyes-open balance (EOB), eyes-closed balance (ECB), standing long jump (SLJ), vertical jump (VJ), dominant 30° knee joint position sense, and non-dominant 45° knee joint position sense (*p* < 0.05), indicating that changes over time differed between the experimental and control groups for these variables. No significant interaction effects were found for dominant 45°, dominant 60°, non-dominant 30°, and non-dominant 60° (*p* > 0.05), suggesting similar changes over time in both groups. A significant main effect of time was observed for EOB, ECB, SLJ, and VJ (*p* < 0.05), while the main effect of group was significant for SLJ and non-dominant 45° (*p* < 0.05).

**Conclusion:**

An 8-week plyometric training program led to significant improvements in balance (EOB, ECB) and explosive performance (SLJ, VJ), as well as in selected knee joint position sense variables (dominant 30° and non-dominant 45°), as indicated by significant group × time interaction effects. No significant effects were observed for dominant 45°, dominant 60°, non-dominant 30°, or non-dominant 60°. The significant interaction observed at the non-dominant 45° angle suggests that changes over time differed between groups, although baseline variability may have contributed to this difference.

**Trial registration:**

Registration date: [29.12.2025] (ClinicalTrials.gov ID: NCT07351539).

**Supplementary Information:**

The online version contains supplementary material available at 10.1186/s13102-026-01762-1.

## Introduction

Plyometric training is one of the training methods aimed at enhancing certain biomotor abilities of athletes, such as speed, strength, and explosive power, and maximizing overall performance levels [1]. The primary objective of this training is to develop the fastest and most explosive muscle groups through the stretch–shortening cycle, thereby elevating the athlete’s current performance capacity to its highest level. Plyometric training is among the most widely used methods for improving performance parameters that are fundamental across all sports disciplines [2, 3]. The literature indicates that plyometric training enhances core motor abilities such as explosive strength, speed, agility, and balance; these effects have also been consistently reported in meta-analyses and systematic reviews [4–7].

Balance can be defined as the ability to control and maintain body position. It is generally examined in two main categories: static and dynamic balance. Static balance refers to the ability to maintain equilibrium in a stationary position, whereas dynamic balance refers to the ability to maintain stability while moving [8, 9]. From an athletic performance perspective, balance directly affects the accuracy, speed, and effectiveness of executed movements. In sports disciplines where balance plays a critical role in performance, a well-developed balance ability is essential for achieving high performance levels [10, 11]. Taekwondo athletes are required to possess superior balance abilities due to continuous changes in body position during kicking techniques and rapid rotational movements [12]. In this context, taekwondo training programs should incorporate specific exercises aimed at improving balance control and postural stability. In addition to specialized training protocols, previous studies have reported that taekwondo training has positive effects on balance, suggesting that regular taekwondo practice enhances balance control by accelerating the development of the vestibular system (inner ear) [13, 14]. Furthermore, dynamic movements commonly performed in taekwondo, such as sudden changes of direction and single-leg jump landings, require a high level of joint position sense (proprioception) awareness [15]. Proprioception is defined as the ability to perceive the position of the body in space and is mediated by sensory information from mechanoreceptors located in the joints, muscles, tendons, and skin [16, 17]. Joint position sense, one of the fundamental components of the proprioceptive system, plays a crucial role in maintaining joint stability during both dynamic movements and static positions [18]. Therefore, plyometric training may be considered an effective training method for improving joint awareness, balance, and neuromuscular properties [19]. Plyometric exercises are described as reactive neuromuscular training methods that stimulate muscle spindles by utilizing the elastic energy stored in the muscle–tendon unit during eccentric loading, thereby enabling maximal force production during the concentric phase [20]. Several studies have reported that plyometric training improves key determinants of athletic performance [21, 22]. Myer et al. [21] reported improvements in balance performance among female high school volleyball players following a seven-week plyometric training program. Similarly, Alikhani et al. [22] investigated the effects of a six-week plyometric training program on dynamic balance and knee proprioception in female badminton players and reported significant improvements in both dynamic balance and knee proprioception after the intervention.

Although the effects of plyometric training on balance, knee joint position sense, and explosive power have been investigated in athletes across different sports disciplines and age groups, studies involving young taekwondo athletes remain limited. Moreover, the existing literature has generally focused on a single gender, and controlled samples with balanced gender distribution are not common. In the present study, the experimental and control groups were composed of female and male athletes in an equal and balanced manner. By examining the effects of an 8-week plyometric training program on multidimensional performance outcomes in young taekwondo athletes, this study aims to address the existing gap in the literature regarding both sample characteristics and methodological design. Accordingly, the aim of this study was to determine the effects of an 8-week plyometric training program on balance, joint position sense, and explosive strength in young taekwondo athletes.

## Materials and methods

### Study design and participants

A total of 20 club-level taekwondo athletes aged between 15 and 18 years voluntarily participated in the study. Participants were randomly divided into two groups: an experimental group consisting of 10 athletes (5 females, 5 males) and a control group consisting of 10 athletes (5 females, 5 males). Inclusion criteria required that participants had no current injuries or any recent injuries that could adversely affect their performance. A pre-test–post-test research design was employed. Following the measurement of anthropometric characteristics (height and body mass), assessments of balance, knee joint position sense, standing long jump, and vertical jump were conducted. After the completion of pre-test measurements, the experimental group performed a plyometric training program in addition to their regular taekwondo training for eight weeks, three times per week (Monday, Wednesday, and Friday), whereas the control group continued only their regular taekwondo training. Post-test measurements were obtained from both groups at the end of the eight-week training period. All assessments were conducted in the performance measurement laboratory of the Faculty of Sport Sciences. This study was conducted in accordance with the decision of the Non-Interventional Clinical Research Ethics Committee of Selçuk University Faculty of Sport Sciences (Approval date: 28.01.2021, Decision number: 09). Furthermore, all participants were informed about the procedures approved by the ethics committee and in compliance with the Declaration of Helsinki, and provided written informed consent prior to participation.

### Trial registration

The trial was registered retrospectively with ClinicalTrials.gov (NCT07351539) due to administrative and procedural timelines related to ethics committee approval and registry processing; however, ethical approval and written informed consent from participants and their parents were obtained prior to participant enrollment and the commencement of the intervention.

### Height measurements

The heights of the volunteer taekwondo athletes were recorded in centimeters (cm). To ensure accuracy and eliminate potential sources of error during height measurement, athletes were measured barefoot and wore only a t-shirt and shorts. Measurements were conducted using an MST-B01 stadiometer.

### Body weight measurements

Measurements were taken twice, as the first and second assessments, using the Tanita BC 601 body composition analyzer. Care was taken to ensure that the device was placed on a flat and hard surface during the measurements. Athletes were barefoot and wore no clothing that could affect their weight. Body weights were recorded in kilograms (kg).

### Balance test

Balance performance was assessed using the Biodex Balance System (Biodex Balance System, BBS; Biodex Medical Systems Inc., Shirley, NY, USA). The system consists of a movable balance platform capable of tilting up to 20° in all directions, allowing a 360° range of motion. The device provides 12 adjustable stability levels, with Level 1 representing the least stable and Level 12 the most stable condition. The platform is connected to dedicated computer software that enables objective evaluation of balance performance, providing an overall postural stability index as well as directional indices in the anteroposterior (AP) and mediolateral (ML) planes. The postural stability index reflects overall balance ability, with higher scores indicating poorer balance performance [23, 24]. Prior to testing, participants were asked to identify their dominant leg. After determining leg dominance, athletes were positioned on the platform standing on the dominant leg with the knee slightly flexed at approximately 45°, while the non-dominant leg was flexed at 90° at the knee. Participants maintained their arms crossed over the chest throughout the test. During the assessment, visual feedback from the screen was eliminated, and athletes were instructed to focus on a fixed point located approximately 1 m away at eye level. For postural control assessment, the platform stability was set at Level 8 under eyes-open (EO) conditions and Level 10 under eyes-closed (EC) conditions. Measurements were conducted under both EO and EC conditions. Prior to testing, the athletes performed three familiarization trials to adapt to the measurement device. Subsequently, balance performance was assessed under eyes-open and eyes-closed conditions, each lasting 30 s. A 5-min rest interval was provided between tests. Pre- and post-training measurements were administered using identical testing procedures [25].

### Knee joint position sense assessment

Knee joint position sense was assessed using an isokinetic dynamometer (HUMAC NORM, Lumex Inc., Ronkonkoma, NY, USA). Prior to testing, the dynamometer settings were individually adjusted according to each participant’s anthropometric characteristics. Measurements were performed for both the dominant and non-dominant legs. Participants were provided with three familiarization trials before data collection to ensure adaptation to the isokinetic dynamometer. During testing, the knee joint of both the dominant and non-dominant limbs was positioned at maximum flexion and then slowly moved toward extension. At flexion angles of 30°, 45°, and 60°, the movement was paused for 10 s at each angle to allow participants to learn the target positions. Subsequently, the knee was returned to maximum flexion, and participants were instructed to actively reproduce the previously learned target angles (30°, 45°, and 60°) by extending the knee. Each angle was tested three times for both the dominant and non-dominant legs. A 2-min rest period was provided between trials. The mean absolute error from the target angles was calculated, and the average deviation was recorded as the knee joint position sense value for both the dominant and non-dominant legs [26, 27].

### Vertical jump test

Vertical jump performance was assessed using a TKK 5406 jump meter. Prior to testing, participants were allowed sufficient practice trials to become familiar with the testing device. During the test, athletes stood on the measurement mat with both feet and were instructed to jump as high as possible at a self-selected time following the visual signal from the device. Arm and leg swing were permitted during the jump. Participants were instructed to land on the mat with both feet simultaneously after the jump. Vertical jump height was displayed on the device screen and recorded immediately after each trial. The test was repeated three times with a 1-min rest interval between trials. The highest value obtained from the three trials was recorded as the participant’s vertical jump performance [28].

### Standing long jump test

Athletes were positioned with their toes just behind the starting line, feet at a normal width, and with their arms extended forward and knees bent in a pre-jump stance. They were instructed to jump as far as possible, using their arms for momentum, and both feet were to land simultaneously. The distance from the starting line to the athlete’s heels was measured in centimeters and recorded. Athletes were given two attempts, and the best result was taken for analysis [29].

### Plyometric training protocol

The program was implemented three days per week for 8 weeks, with each training session lasting approximately 30 min. Box and hurdle heights were set at 40 cm. The bounding distance progressed from approximately 5 m at the beginning to 7–8 m over time, thereby preserving technical integrity while supporting adaptations in explosive power. As reported in the literature, maintaining controlled box and hurdle heights in young athletes reduces the risk of injury and maximizes performance development [4, 30–35].

**Table:** Plyometric training program (Weeks 1–2)

MONDAY	WEDNESDAY	FRIDAY	Sets * Repetitions	Rest Between Repetitions – Rest Between Sets
Squat Jump	Squat Jump	Squat Jump	3*5	30–90 s
Jump to Box	Jump to Box	Jump to Box	3*10	30–90 s
Lateral Jump to Box	Lateral Jump to Box	Lateral Jump to Box	3*10	30–90 s
Bounding With Rings	Bounding With Rings	Bounding With Rings	2*12	30–90 s
Lateral Hurdle Jump	Lateral Hurdle Jump	Lateral Hurdle Jump	2*15	30–90 s
Tuck Jump	Tuck Jump	Tuck Jump	3*10	30–90 s
TRAINING CONTENT			TRAINING DURATION 30 MIN	

**Table:** Plyometric training program (Weeks 3–4)

MONDAY	WEDNESDAY	FRIDAY	Sets * Repetitions	Rest Between Repetitions – Rest Between Sets
Split Squat Jump	Split Squat Jump	Split Squat Jump	3*10	30–90 s
Tuck Jump	Tuck Jump	Tuck Jump	3*10	30–90 s
Lateral Box Push Offs	Lateral Box Push Offs	Lateral Box Push Offs	3*10	30–90 s
Jump to Box	Jump to Box	Jump to Box	3*12	30–90 s
Lateral Hurdle Jump	Lateral Hurdle Jump	Lateral Hurdle Jump	2*15	30–90 s
Bounding	Bounding	Bounding	3*5	30–90 s
TRAINING CONTENT			TRAINING DURATION 30 MIN	

**Table:** Plyometric training program (Weeks 5–6)

MONDAY	WEDNESDAY	FRIDAY	Sets * Repetitions	Rest Between Repetitions – Rest Between Sets
Tuck Jump	Tuck Jump	Tuck Jump	3*12	30–90 s
Single Leg Lateral Hops	Single Leg Lateral Hops	Single Leg Lateral Hops	3*12	30–90 s
Split Squat Jump	Split Squat Jump	Split Squat Jump	3*10	30–90 s
Lateral Jump to Box	Lateral Jump to Box	Lateral Jump to Box	3*10	30–90 s
Zig Zag Hops	Zig Zag Hops	Zig Zag Hops	5*2	30–90 s
Lateral Hurdle Jump	Lateral Hurdle Jump	Lateral Hurdle Jump	2*15	30–90 s
TRAINING CONTENT			TRAINING DURATION 30 MIN	

**Table:** Plyometric training program (Weeks 7–8)

MONDAY	WEDNESDAY	FRIDAY	Sets * Repetitions	Rest Between Repetitions – Rest Between Sets
Tuck Jump	Tuck Jump	Tuck Jump	3*12	30–90 s
Squat Jump	Squat Jump	Squat Jump	3*10	30–90 s
Single Leg Lateral Hops	Single Leg Lateral Hops	Single Leg Lateral Hops	3*12	30–90 s
Split Squat Jump	Split Squat Jump	Split Squat Jump	3*10	30–90 s
Lateral Hurdle Jump	Lateral Hurdle Jump	Lateral Hurdle Jump	2*15	30–90 s
Jump to Box	Jump to Box	Jump to Box	3*12	30–90 s
TRAINING CONTENT			TRAINING DURATION 30 MIN	

### Statistical analysis

Descriptive statistics are presented as mean ± standard deviation (Mean ± SD). The normality of the data distribution was assessed using the Shapiro–Wilk test. The study followed a 2 × 2 mixed-design (two groups × two time points) experimental design. Accordingly, a two-way repeated measures ANOVA (mixed-design ANOVA) was performed to examine the main effects of group (experimental vs. control) and time (pre-test vs. post-test), as well as the group × time interaction effect. When a significant interaction effect was observed, pairwise comparisons were conducted using Bonferroni-adjusted post hoc tests. Effect sizes were reported as partial eta squared (η²p) and interpreted according to Cohen’s [36] criteria, where 0.01, 0.06, and 0.14 indicate small, medium, and large effect sizes, respectively. Statistical significance was set at *p* < 0.05. All statistical analyses were performed using IBM SPSS Statistics (Version 27.0, IBM Corp., Chicago, IL, USA).

An a priori power analysis was conducted using GPower software (version 3.1) for a mixed-design repeated-measures ANOVA (within–between interaction). Assuming a moderate-to-large effect size (f = 0.35), an alpha level of 0.05, and a desired power of 0.80, with two groups and two repeated measurements, the required minimum sample size was estimated as 20 participants.

## Results

The descriptive characteristics of the participants are presented in Table 1.

**Table 1:** Descriptive characteristics of the participants (*n* = 10)

Variables	Experimental Group	Control Group
	Mean ± SD	Mean ± SD
Age (year)	16.90 ± 0.74	16.90 ± 1.20
Height (cm)	169.00 ± 10.58	174.30 ± 6.20
Body Weight (kg)	55.50 ± 7.08	65.40 ± 12.76
Sport Experience (year)	6.50 ± 1.35	6.30 ± 1.34

According to Table 2, significant group × time interaction effects were observed for EOB (*p* < 0.001, η²*p* = 0.646), ECB (*p* = 0.002, η²*p* = 0.429), SLJ (*p* < 0.001, η²*p* = 0.615), VJ (*p* < 0.001, η²*p* = 0.713), dominant 30° (*p* = 0.039, η²*p* = 0.215), and non-dominant 45° (*p* = 0.006, η²*p* = 0.354), indicating that changes over time differed between the experimental and control groups for these variables. For dominant 45°, non-dominant 30°, and non-dominant 60° knee joint position sense, no significant interaction effects were found (*p* > 0.05), suggesting similar changes over time in both groups. A significant main effect of time was observed for EOB, ECB, SLJ, and VJ (*p* < 0.05), while the main effect of group was significant only for SLJ and non-dominant 45° (*p* < 0.05).

**Table 2:** Main effects over time and between group for experimental group and control group

Variables	Groups	Pre-Test (Mean ± SD)	Post-Test (Mean ± SD)	Time *p*	ES	Group *p*	ES	Group×Time *p*	ES
EOB	Experimental	2.25 ± 0.50	1.67 ± 0.44	< 0.001	0.735	0.062	0.181	< 0.001	0.646
	Control	2.71 ± 1.12	2.65 ± 0.98						
ECB	Experimental	3.09 ± 0.97	2.52 ± 0.84	0.001	0.481	0.324	0.054	0.002	0.429
	Control	3.17 ± 0.78	3.14 ± 0.47						
SLJ (cm)	Experimental	200.70 ± 21.58	215.10 ± 24.34	< 0.001	0.621	0.022	0.260	< 0.001	0.615
	Control	181.20 ± 25.46	181.30 ± 23.88						
VJ (cm)	Experimental	54.00 ± 8.18	58.40 ± 7.59	< 0.001	0.731	0.058	0.186	< 0.001	0.713
	Control	48.20 ± 9.66	48.30 ± 9.59						
Dominant 30°	Experimental	1.40 ± 0.60	0.53 ± 0.36	0.559	0.019	0.002	0.409	0.039	0.215
	Control	1.73 ± 1.30	2.23 ± 1.17						
Dominant 45°	Experimental	2.10 ± 0.97	1.50 ± 1.33	0.089	0.152	0.439	0.034	0.775	0.005
	Control	2.43 ± 1.76	2.00 ± 1.19						
Dominant 60°	Experimental	2.27 ± 1.61	1.17 ± 1.20	0.054	0.191	0.251	0.073	0.517	0.024
	Control	2.53 ± 1.34	1.97 ± 1.21						
Non-dominant 30°	Experimental	2.70 ± 1.47	1.67 ± 1.03	0.101	0.142	0.818	0.003	0.354	0.048
	Control	2.50 ± 1.79	2.20 ± 2.60						
Non-dominant 45°	Experimental	2.30 ± 1.33	1.20 ± 0.82	0.547	0.020	0.049	0.199	0.006	0.354
	Control	2.37 ± 1.73	4.00 ± 2.75						
Non-dominant 60°	Experimental	2.17 ± 1.87	1.50 ± 0.95	1.000	0.000	0.220	0.082	0.079	0.161
	Control	2.13 ± 1.08	2.80 ± 1.40						

## Discussion

This study was conducted to determine the effects of an 8-week plyometric training program on balance, joint position sense, and explosive strength in young taekwondo athletes. Overall, the findings indicate that plyometric training has positive effects on balance, dominant 30°, non-dominant 45° knee joint position sense, standing long jump, and vertical jump performance.

Improvements in balance were supported by significant group × time interaction effects, indicating that the experimental group improved more than the control group. The positive effects of plyometric training on balance are largely explained by neuromuscular adaptations. Markovic ve Mikulic [37] reported that plyometric training increases motor unit activation, improves the elastic properties of the muscle–tendon unit, and shortens reflex response times. These adaptations enable faster and more effective muscular responses to sudden losses of balance. In addition, the effective use of the stretch-shortening cycle (SSC) reduces the transition time between eccentric and concentric contractions of the lower-extremity muscles, thereby contributing to the maintenance of postural stability. In this study, an 8-week plyometric training program was found to improve balance performance in young taekwondo athletes. The present findings are consistent with a substantial body of literature reporting beneficial effects of plyometric training on balance [38–42]. Ramírez-Campillo et al. [38] examined the effects of 6 weeks of vertical, horizontal, and combined plyometric training in young soccer players and reported improvements in balance performance across different training modalities. Jlid et al. [39] also reported that a 6-week multidirectional plyometric training program enhanced balance performance in young soccer players. Asadi ve Arazi [40] demonstrated that 6 weeks of plyometric training significantly improved balance in young basketball players Bouteraa et al. [41] reported improvements in postural control following 8 weeks of plyometric training in female basketball players. Similarly, Cherni et al. [42] investigated the effects of an 8-week in-season plyometric training program in elite female basketball players and reported improvements in both static and dynamic balance performance. Overall, an 8-week training period appears to be sufficient for improving balance performance. The literature suggests that 6–8 weeks of plyometric training is adequate to induce neuromuscular adaptations. In this regard, the duration of the present study is consistent with previous research and provides an explanation for the significant improvements observed.

Significant group × time interaction effects were observed at the dominant 30° and non-dominant 45° angles, indicating that changes over time differed between the experimental and control groups. These findings suggest that proprioceptive adaptations following plyometric training may be angle-specific. Joint position sense is critically important in combat sports for the accurate angular control of complex lower-extremity movements. In taekwondo techniques, kick height, speed, and accuracy largely depend on knee joint position sense. Moreover, the improvements observed in selected knee joint angles in young taekwondo athletes may reflect partial bilateral adaptations of the training intervention, suggesting that the central nervous system may regulate inter-limb coordination more efficiently following training. The relatively large effect size observed at the non-dominant 45° angle suggests that plyometric training induces meaningful neuromuscular adaptations, particularly in proprioceptive control. In contrast, no significant interaction effects were observed at the dominant 45°, dominant 60°, non-dominant 30°, and non-dominant 60° angles, indicating similar changes over time in both groups.

During eccentric loading, muscle spindles and joint receptors receive high levels of stimulation, which contributes to the reorganization of proprioceptive sensitivity in the sensorimotor cortex [43]. In addition, increased tension in ligaments and tendons during the rapid eccentric–concentric cycle enhances the sensitivity of Pacinian corpuscles and Ruffini endings, thereby improving joint position sense [44–46]. Asadi et al. [47] reported positive effects of plyometric training on knee joint angular accuracy. Zamani et al. [48] found a significant improvement in proprioceptive error at 30° and 45° knee angles following 8 weeks of plyometric training. Marks and Quinney [49] stated that mid-range flexion angles (approximately 45°) provide higher sensitivity for proprioceptive assessment. In this context, the significant improvement observed at the non-dominant 45° angle in the present study is consistent with the biomechanical optimization of proprioceptive sensitivity. These findings are in line with evidence indicating that proprioception is trainable and also support literature emphasizing angle-dependent effects [49, 50]. Although improvements were observed bilaterally in the present study, the more pronounced improvements in the non-dominant limb suggest that plyometric loading can induce bilateral neuromuscular adaptations.

Significant group × time interaction effects observed for standing long jump (SLJ) and vertical jump (VJ) indicate that improvements over time were greater in the experimental group compared to the control group. These results strongly align with the existing literature emphasizing the effects of plyometric training on lower-extremity explosive power capacity [4, 30, 40, 51–53].

Maffiuletti et al. [30] reported that a 4-week plyometric training program integrated into preseason volleyball training significantly increased vertical jump height. Asadi ve Arazi [40] also found significant improvements in both vertical jump and standing long jump performance following 6 weeks of high-intensity plyometric training in young basketball players. Meta-analytical studies have similarly reported that plyometric training improves vertical jump performance [4, 51]. Vassil and Bazanovk [52] described greater increases in vertical jump performance in volleyball players who performed additional plyometric training compared with those who only followed regular training. Singh et al. [53] also demonstrated that 6 weeks of plyometric training improved vertical jump performance in taekwondo athletes. Although previous studies have consistently shown that plyometric training improves vertical jump and standing long jump performance, the present study differs from earlier research in terms of training protocol, duration, and participant characteristics, providing a distinct experimental design within the literature.

### Limitations

This study has several limitations that should be acknowledged. The relatively small sample size may restrict the statistical power of the analyses and limit the generalizability of the findings to a broader athletic population. Although the participants were selected based on strict inclusion criteria and represent a specific group of trained taekwondo athletes, future studies with larger sample sizes are needed to enhance external validity.

Future research should include formal power calculations to determine the optimal sample size and strengthen the robustness of statistical findings.

Despite these limitations, the study provides valuable preliminary evidence regarding the effects of plyometric training on performance and proprioceptive outcomes in young taekwondo athletes.

## Conclusion

In conclusion, significant group × time interaction effects were observed for balance (EOB, ECB), explosive performance (SLJ, VJ), and dominant 30° knee joint position sense, indicating that changes over time differed between the experimental and control groups for these variables. In contrast, no significant interaction effects were found for dominant 45°, non-dominant 30°, and non-dominant 60° knee joint position sense, suggesting similar changes over time in both groups. For non-dominant 45° knee joint position sense, a significant interaction effect was observed; however, the presence of baseline differences between groups suggests that this finding should be interpreted with caution and may not be attributed solely to the training intervention. Overall, the findings indicate that an 8-week plyometric training program produces improvements in balance, explosive performance, and selected proprioceptive outcomes, while its effects on joint position sense appear to be angle-specific.

## Supplementary Information

Supplementary Material 1.

Supplementary Material 2.

## Data Availability

The datasets used and/or analyzed during the current study are available from the corresponding author on reasonable request.

## Abbreviations

BBS: Biodex balance system
Cm: Centimeter
Min: Minute
SLJ: Standing long jump
VJ: Vertical jump
EO: Eyes open
EC: Eyes closed
EOB: Eyes open balance
ECB: Eyes closed balance
Kg: Kilogram
