# Supplementary material for: Effects of plyometric training on balance, joint position sense, and explosive strength in young taekwondo athletes
Source: BMC Sports Sci Med Rehabil. 2026 May 22;18:327. doi: 10.1186/s13102-026-01762-1 (PMC13371514; doi:10.1186/s13102-026-01762-1)
Supplement: Supplementary file 1 — Supplementary Material 1. [file 13102_2026_1762_MOESM1_ESM.pdf]

Figure 1: CONSORT_2025_filled_SIR_SSG

Flow diagram of the progress through the phases of a randomised trial of two groups (that is, enrolment, intervention allocation, follow-up, and data analysis)

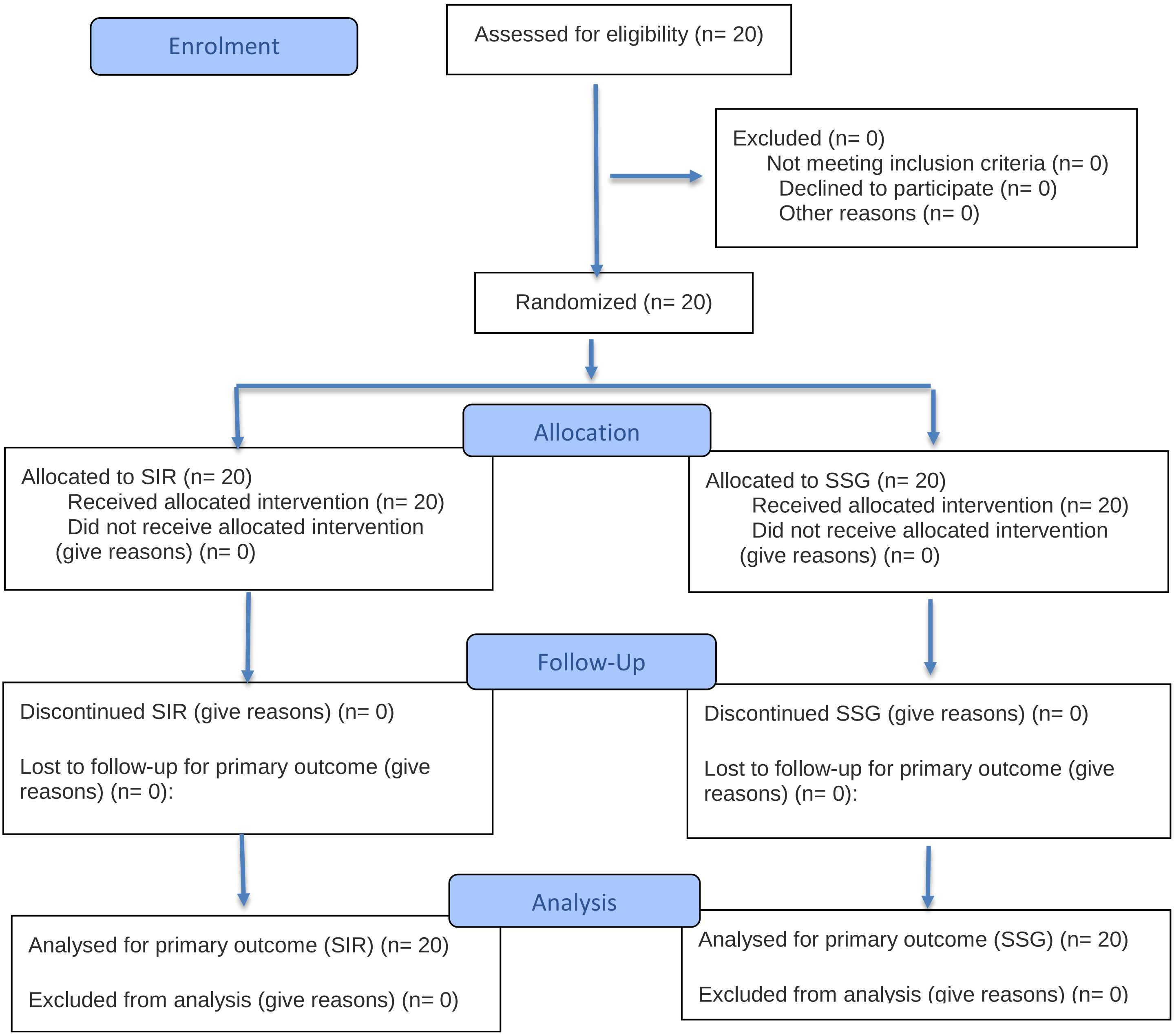

Citation: Hopewell S, Chan AW, Collins GS, Hróbjartsson A, Moher D, Schulz KF, et al. CONSORT 2025 Statement: updated guideline for reporting randomised trials. BMJ. 2025; 388:e081123. https://dx.doi.org/10.1136/bmj-2024-081123

© 2025 Hopewell et al. This is an Open Access article distributed under the terms of the Creative Commons Attribution License (https://creativecommons.org/licenses/by/4.0/), which permits unrestricted use, distribution, and reproduction in any medium, provided the original work is properly cited.
